# Pressure prescription in blood flow restriction training: evaluating the role of arterial occlusion pressure

**DOI:** 10.3389/fspor.2026.1856910

**Published:** 2026-06-17

**Authors:** Joel Novak

**Affiliations:** Department of Physical Therapy, Quincy University, Quincy, IL, United States

**Keywords:** AOP, arterial occlusion pressure, BFR, blood flow restriction, limb occlusion pressure, LOP

## Abstract

Blood flow restriction (BFR) training has consistently demonstrated the capacity to elicit meaningful physiological adaptations across clinical, rehabilitative, and performance contexts. Central to BFR application is the concept of balancing vascular restriction with exercise demands in efforts to support both safety and effectiveness. Within this framework, individualizing restrictive pressure relative to arterial occlusion pressure (AOP) has been advanced as a strategy to standardize dosing across devices, limb characteristics, and populations. Although AOP-based prescription provides a structured and mechanistically grounded approach, its ability to fully characterize the internal physiological stimulus of BFR remains an area of ongoing discussion. AOP is influenced by cuff characteristics, limb morphology, vascular responsiveness, body position, and exercise conditions, introducing variability into its interpretation. Consequently, resting arterial occlusion values may not fully represent the dynamic metabolic and hemodynamic environment present during exercise. While AOP-based models offer advantages for standardization, consistent superiority in outcomes has not been demonstrated across contexts. This narrative review synthesizes contemporary BFR literature with specific attention to the assumptions underlying AOP-centered prescription strategies. Current evidence suggests that individualized AOP measurement is not universally required to achieve safe and effective adaptations when broader programming variables and clinical oversight are appropriately managed. Significant adaptations occur across a spectrum of well-tolerated pressures, including those derived from AOP-based approaches. Situating pressure determination within a broader, outcome-oriented framework may better reflect the multifactorial nature of BFR physiology and support continued refinement of both research methodology and clinical implementation.

## Introduction

Blood flow restriction (BFR) training has become a prominent intervention across rehabilitation, sports medicine, and performance settings (1–7). As BFR utilization has expanded, attention has increasingly turned toward optimizing its prescription, particularly with respect to pressure selection. Among the proposed approaches, arterial occlusion pressure (AOP) has gained prominence as a method for individualizing cuff inflation pressures. AOP, also referred to as limb occlusion pressure (LOP), is defined as the minimum external cuff pressure required to completely stop arterial blood flow to a limb at rest (8–10). Relative pressures expressed as a percentage of AOP are commonly used in experimental protocols and are frequently presented as a means of standardizing stimulus across participants while accounting for inter-individual variability in limb size, tissue composition, and cuff characteristics (8, 11–14). AOP has shown safe and successful results throughout the available literature (11–14). As such, AOP represents a defensible and widely accepted method of BFR application within both research and clinical contexts.

As the use of AOP-based prescription has expanded, continued evaluation of its conceptual role in BFR dosing appears warranted. Although AOP provides a reproducible vascular reference point, its role as a proxy for training dose, physiological stress, or adaptive stimulus has not been definitively established. Framing personalization primarily through AOP-based prescription may not fully capture the multifactorial determinants of BFR responses (7, 12, 13, 16–18).

The purpose of this narrative review is not to dismiss the value of AOP-based methods, nor to suggest that such approaches are not beneficial. Rather, this review aims to critically synthesize the existing literature to clarify the extent to which AOP meaningfully explains variability in BFR outcomes with the goal of equipping users and clinicians with a more comprehensive understanding of devices, pressure protocols, safety considerations and physiological adaptations. Importantly, this review explicitly distinguishes between the concepts of necessity, benefit, and methodological utility. By examining historical context, mechanistic evidence, and outcome-based data across devices and populations, the evidence supports methodological utility and benefit with AOP-based prescriptions; however, it challenges AOP's elevation as a necessary or singular determinant of effective BFR dosing. This review considers reframing personalization in BFR as a construct that extends beyond a single vascular metric.

## Methodology

This manuscript was conducted as a narrative review to examine whether arterial occlusion pressure (AOP) is necessary for the safe and effective application of BFR in clinical, performance, and rehabilitation contexts. A narrative review methodology was selected to allow for conceptual synthesis and integration of heterogeneous evidence spanning physiology, clinical outcomes, device engineering, and implementation practices, where methodological variability limits the feasibility of meta-analytic pooling.

A structured literature search was conducted across commonly used databases in rehabilitation and sports medicine including APTA Article Search, PubMed/Medline, CINAHL, and ScienceDirect (2000–2026). Search terms were combined using Boolean operators and included combinations of blood flow restriction (BFR/BFRT/pBFR), occlusion/limb occlusion pressure (AOP/LOP), cuff/restrictive pressure, low-load resistance training, safety/adverse events, and clinical application. Reference lists of key reviews and foundational studies were manually screened to identify additional relevant publications.

Literature was screened by title/abstract for relevance to BFR application, pressure prescription, physiological response, safety, or clinical outcomes. Full texts were included if they contributed to understanding how restrictive pressure is determined, justified, applied, or interpreted. No restrictions were placed on study design.

**Figure 1:**
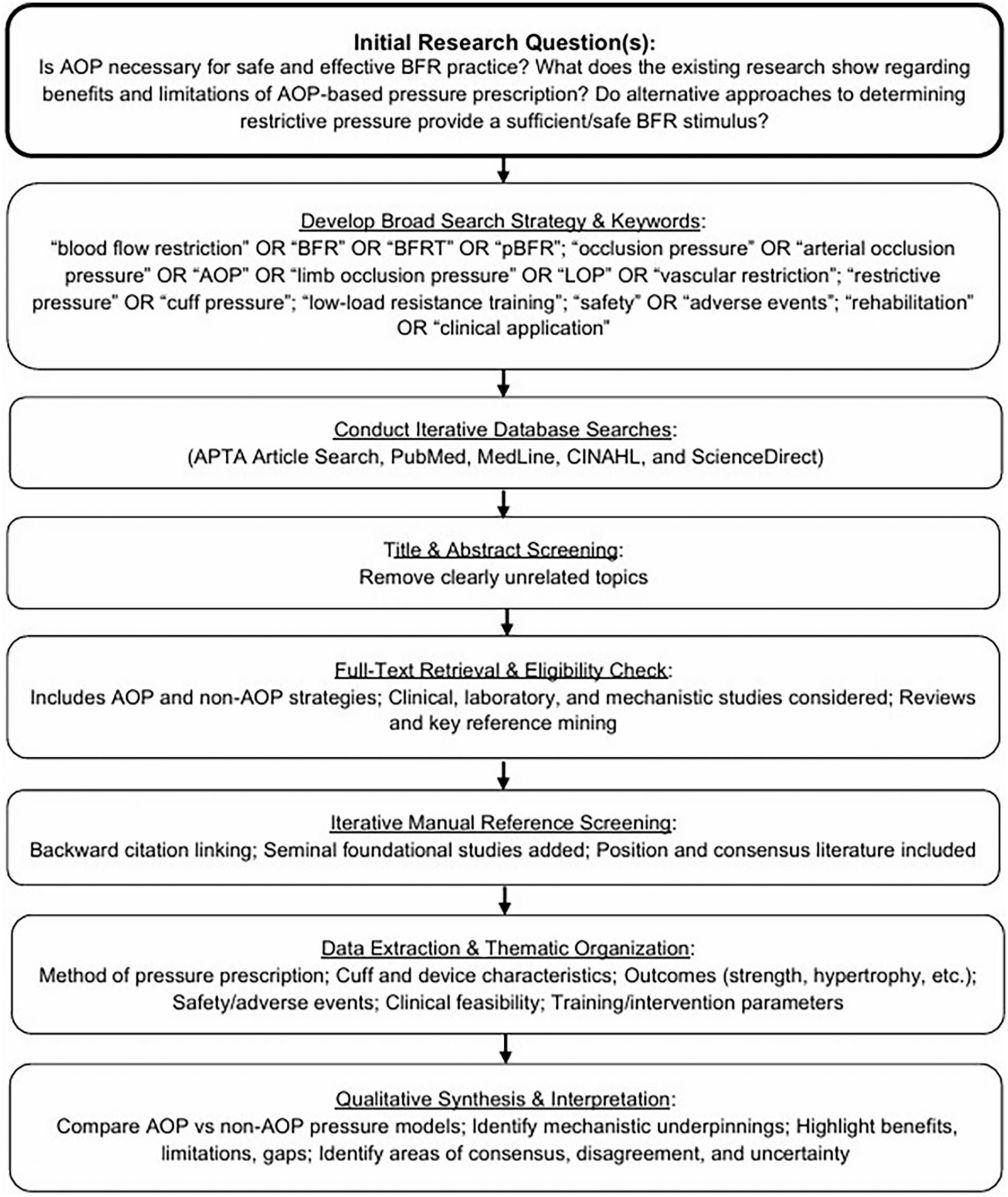
Conceptual flow diagram illustrates the methodology employed in this narrative review. The framework highlights an iterative process of literature search refinement, thematic categorization, and qualitative synthesis designed to address the central question concerning the necessity of arterial occlusion pressure (AOP) in blood flow restriction (BFR) practice. Core stages include development of a comprehensive search strategy, targeted database queries, manual reference screening, structured thematic data extraction, conceptual synthesis, and iterative manuscript refinement.

Rather than formal data pooling, information was extracted qualitatively and organized thematically (AOP-based vs. non-AOP-based pressure determination; device characteristics; intervention parameters; physiological responses; outcomes; safety; feasibility). Interpretation emphasized conceptual coherence, methodological transparency, and clinical relevance. As a narrative review, this approach is inherently subject to selection bias and interpretive judgment; to mitigate these limitations, inclusion was broad, search strategy was transparent, and contrasting findings were intentionally incorporated. The literature search strategy, thematic categorization process, and qualitative synthesis framework illustrated in Figure 1 were used throughout this review.

## Background

### The evolution of BFR prescription and the emergence of AOP

Early BFR investigations demonstrated that low-load resistance exercise performed under conditions of partial vascular restriction could elicit increases in muscle size and strength comparable to traditional high-load training (19, 21, 55–57). Importantly, many of these foundational studies employed fixed absolute pressures or loosely-defined pressure ranges rather than individualized occlusion measurements, yet still produced safe, consistent and clinically meaningful adaptations.

As the BFR literature expanded, methodological refinement became a central focus. The introduction of AOP-based prescription represented a logical attempt to improve experimental control by anchoring training pressures to an individualized vascular reference point (10, 16). Expressing cuff pressures as a percentage of AOP was proposed as a means of reducing inter-subject variability, enhancing internal validity within laboratory studies, and avoiding supra-occlusion (which is commonly viewed as a modifiable safety consideration) (8, 9). Notably, AOP has shown that it offers advantages for experimental control, internal validity, and methodological consistency, particularly in mechanistic and comparative trials (12, 14, 18, 22, 23).

Over time, however, the scope of AOP's perceived importance broadened. What initially served as a methodological standardization tool has increasingly been interpreted as evidence that AOP is a necessary or superior determinant of effective BFR dosing. Advances in cuff technology and automated pressure regulation systems have further emphasized vascular calibration as a component of individualized prescription. Importantly, the absence of demonstrated superiority should not be interpreted as evidence of inferiority. Rather, the literature to date suggests a lack of clear outcome-based differentiation between AOP-based and alternative pressure prescription strategies (12, 18, 24–27).

Despite growing advocacy for AOP-based regulation, findings from both historical and contemporary research indicate that the current evidence supports a more nuanced interpretation of pressure prescription strategies. Effective BFR outcomes have been observed across a wide range of pressures, cuff widths, and device types, many of which do not rely on direct measurement of arterial occlusion (7, 12, 16, 17, 24, 26).

## Physiology

### Mechanisms underpinning BFR adaptations

It is well-established that the physiological impact of a given restrictive pressure is strongly modulated by factors such as cuff width, shape, material composition, body position, limb circumference, time of day, and the activity being performed/studied (10, 20, 23, 28–35). The use of individualized pressures based on arterial occlusion pressure (AOP) has been widely proposed as a means of optimizing this balance and standardizing application across devices, limb sizes, and populations. AOP-guided protocols are well supported and have produced safe and effective outcomes. Notably, AOP-based evidence has demonstrated benefit across a broad range of restrictive pressures (12, 18, 35–37).

These findings highlight the importance of considering how AOP itself is conceptualized within the context of BFR exercise. Arterial occlusion pressure should be interpreted as a transient physiological measurement obtained under specific resting conditions rather than a fixed hemodynamic constant. In contrast, BFR exercise is inherently dynamic, involving repeated muscle contractions, fluctuations in intramuscular pressure, changes in limb position, and evolving cardiovascular and metabolic responses that collectively influence limb blood flow during activity (8, 10, 17). Extrapolating resting AOP values to active exercise conditions therefore introduces uncertainty, as the vascular environment during contraction differs meaningfully from that observed at rest (22, 32). Additional influences of limb position, contraction intensity, exercise modality, tissue characteristics, training status, and perceptual tolerance may further contribute to ‘inter’ and ‘intra’ individual variability that is not captured by a resting occlusion threshold (11, 35). Consistent with this, Lixandrão et al. (9) found that occlusion pressure appeared secondary to exercise intensity when higher training intensities were used. Taken together, the available evidence suggests that although AOP offers a standardized reference for prescribing restrictive pressure, adaptive responses to BFR exercise are likely influenced by multiple interacting factors beyond the resting occlusion threshold alone.

Important to this concept is evidence showing that applying a given percentage of AOP does not translate proportionally to an equivalent percentage reduction in arterial inflow, as the pressure-flow relationship appears non-linear (22, 32, 37, 38). For example, although pressures ranging from 40%–80% of AOP are frequently recommended, investigations have demonstrated that this range may result in a relatively similar reduction in limb blood flow of approximately 50%–55%, rather than a linear reduction (1). Thus, the magnitude of arterial inflow restriction may remain comparatively stable across a broad span of prescribed relative pressures, which may help explain reported benefits across wide pressure ranges.

While the relationship between applied pressure and arterial inflow appears non-linear, it is important to distinguish this from downstream physiological consequences of vascular restriction. In particular, venous outflow may be progressively restricted across increasing pressures, contributing to metabolite accumulation, local hypoxia, and elevations in blood lactate that are commonly associated with BFR exercise (17, 19, 28). Measures such as muscle oxygen saturation (SmO₂), heart rate, and circulating lactate have been shown to increase during BFR, reflecting the integrated metabolic and cardiovascular response to restricted flow. However, these responses demonstrate substantial inter-individual variability and do not appear to scale in a simple or predictable manner with percentage of AOP alone (22, 32, 37). Accordingly, although higher pressures may augment certain markers of metabolic stress, this does not consistently translate to superior adaptations, reinforcing the importance of interpreting pressure within a broader physiological and programmatic context.

Other investigations have reported mixed findings, suggesting the presence of a lower boundary where insufficient restriction may fail to elicit an adequate adaptive stimulus (9, 39, 40). Alternative studies indicate diminished value and greater perceived discomfort at higher restrictive values, suggesting an upper boundary may exist (16, 18, 22, 37). Collectively, this body of evidence indicates that although a minimum pressure threshold may be required to significantly alter hemodynamic responses, increases beyond an optimal range do not appear to elicit proportionally greater physiological adaptations. In this context, AOP may offer a practical reference point for establishing lower and upper boundaries of restriction, rather than a definitive determinant of training dose.

Mladen et al. (41) conducted a methodologically rigorous study which challenged prevailing assumptions about the dose-response relationship between AOP-applied cuff pressure and physiological adaptation at rest and during exercise. They found that although cardiac output initially declined during BFR, it rapidly returned to baseline via compensatory mechanisms. Notably, despite standardized restriction at 40% AOP, reductions in blood flow varied widely among participants (25%–50%), underscoring substantial interindividual variability and autoregulatory capacity. These findings suggest that fixed AOP prescriptions may not uniformly predict in-exercise physiological responses.

Additional recent evidence highlights the difficulty of maintaining precise AOP during activity, with three out of four commonly used AOP-based cuffs often failing to sustain targeted pressures during rest or exercise (58). Because exercise-induced vasodilation and muscle contractions alter inflow dynamics (and do so differently across individuals), resting AOP may not fully reflect physiological conditions during dynamic exercise (22, 41). Rolnick et al. (35) also reported that physiological responses may vary between cuffs despite standardization to a percentage of AOP.

Overall, cumulative variability underscores the complexity of BFR pressure prescription and suggests that effective dosing likely spans a broad range, even when individualized to AOP. Although AOP-based calibration is often advocated to enhance precision and reduce inter-individual differences, empirical support for superior standardization or outcome optimization remains limited. Adaptations are influenced by interacting variables beyond occlusion percentage, limiting the capacity of AOP alone to define or predict stimulus delivery.

## Safety

### Considerations across BFR pressure prescription strategies

Safety is commonly presented as a rationale for determining arterial occlusion pressure. Large scale surveys, systematic reviews, and consensus documents report a low incidence of serious adverse events across a range of BFR prescription approaches, including those that do not incorporate individualized AOP assessment (2, 12, 35, 36, 42, 56, 57).

Beyond considerations of overt safety, AOP measurements attempt to control for pressure magnitude, which may also influence perceptual and cardiovascular responses during exercise. Multiple studies show that higher restrictive pressures do not consistently produce greater strength or hypertrophy adaptations and may be associated with increased discomfort and cardiovascular strain without clear additive benefit (16, 18, 40). Higher applied pressures have also been shown to augment the exercise pressor reflex, resulting in greater elevations in blood pressure during cuff inflation compared with lower pressure conditions (43, 44). Notably, in the study by Yamada et al., systolic and diastolic blood pressure returned to levels comparable to control conditions immediately following cuff deflation, suggesting that these responses are transient. Nevertheless, the acute hemodynamic stress observed during inflation may warrant consideration in individuals with cardiovascular compromise. In this context, individualized pressure determination, including AOP-based approaches, may assist clinicians in selecting conservative and context-appropriate restrictive pressures when working with higher-risk populations.

As safety considerations are often framed around the avoidance of full arterial occlusion, AOP determination has been elevated as a principal safeguard. Yet, current evidence does not establish a causal relationship between transient supra-occlusive pressures and clinically meaningful harm during exercise (10, 12). Supra-occlusive pressures are not physiologically necessary nor conceptually aligned with the mechanistic rationale of BFR. Therefore, while AOP assessment may contribute to risk management and may serve as a practical starting reference for initial pressure selection when available, it should be interpreted as one component within a broader safety framework rather than a singular protective requirement.

Accordingly, risk mitigation considerations extend beyond occlusion pressure alone. Conservative loading strategies, limits on time under occlusion, appropriate exercise selection, symptom monitoring, and population-specific screening collectively appear to exert substantial influence on safety outcomes (10, 12, 29, 35, 36, 42). Device architecture may also meaningfully shape safety profiles, as cuff width, material properties, and system design influence pressure distribution and vascular response (10, 12, 29, 35, 45). In this context, safety is best conceptualized as emerging from system-level constraints, including protocol design, equipment characteristics, and clinical oversight, rather than from precision pressure targeting in isolation.

While AOP calibration represents a structured and defensible method for individualizing restrictive pressure, particularly when using devices capable of full arterial occlusion, current evidence does not indicate that AOP determination is universally required to establish safe practice. Emphasizing AOP as a singular safeguard may risk overattributing safety to numerical pressure targets, whereas neurological symptoms, exaggerated pressor responses, and discomfort can occur across a range of pressures and are influenced by cuff characteristics, duration of application, and individual tolerance (35, 42, 46). As a result, pressure determination is most appropriately situated within a comprehensive clinical framework in which load management, supervision, screening, and real-time monitoring collectively contribute greater influence on adverse event risk than any single prescription variable.

## Outcome-based evidence

### The limits of pressure precision

Another central issue in the AOP discourse is the assertion that pressure calibration enhances outcomes. It should be acknowledged that AOP-based prescription remains advantageous for internal validity, particularly in mechanistic or comparative trials where controlling vascular exposure is methodologically desirable (10, 12, 14).

While AOP improves internal consistency within experimental designs, its superiority in producing meaningful adaptations has not been conclusively established across populations or training contexts (12, 16–18, 26, 47). Even advanced autoregulated devices that continually adjust to AOP fluctuations during activity have not demonstrated superior safety or efficacy in acute nor long-term outcomes (24, 25, 27, 48, 49), and their cost and complexity may further limit practical use in clinical or general fitness settings.

To this point, Dancy et al. (26) compared three distinct BFR systems employing differing mechanisms to restrict blood flow and found no advantage of auto-regulated over fixed pressure in safety or outcomes. Practical BFR (pBFR), often employing elastic wraps without AOP calibration, is another approach that has demonstrated benefits across diverse populations. Studies report improvements in squat strength in adolescents (50), running speed in young adults (51), VO2 max in elite rowers (52), evidence of bone healing in a bodybuilder (21), and muscle hypertrophy (51). Additional work supports limb circumference–based application of elastic bands as both safe and valid (42, 53). Stray-Gundersen et al. also compared elastic bands with wide-rigid cuffs during walking and reported fewer cardiovascular responses with elastic bands, suggesting potential for conservative application in at-risk populations (34). Indicators such as tolerance, performance, fatigue progression, and symptom response, as well as physiological markers including heart rate elevation, muscle oxygen desaturation, and blood lactate (when available), may provide actionable feedback during sessions irrespective of specific occlusion pressure (15, 17).

This broad efficacy underscores the practicality of BFR across clinical and performance contexts, even in studies where individualized pressure calibration was not employed. The consistent benefits observed under variable pressure conditions suggest that while personalized occlusion pressures hold conceptual and methodological value, generalized application approaches can elicit comparable adaptations without compromising safety.

Importantly, acknowledging these limitations does not diminish the methodological strengths of AOP. Rather, it highlights the need to distinguish between its value for standardization for research purposes and its optimization for clinical outcomes, a distinction not always explicitly addressed in the literature.

## Personalization in BFR

### Conceptual and practical considerations

Personalization is frequently cited as a primary justification for AOP-based BFR prescription. The call for personalization in BFR reflects broader evidence-based frameworks emphasizing individualized decision-making. AOP-based application was developed, in part, to improve reproducibility and guide dosing. Determining BFR pressures based on an individual's arterial occlusion threshold is thought to provide a more personalized, safer, and more consistent stimulus (5, 10, 12, 54). While AOP does offer a reproducible reference point for guiding applied pressures across participants, assumptions of predictable and consistent stimulus have not been empirically confirmed when comparing outcomes to non-AOP devices.

For example, current literature shows that identical percentages of AOP do not produce equivalent physiological responses across individuals. Considerable variability has been documented in blood flow reduction, discomfort, fatigue, and neuromuscular activation at the same relative pressures (16, 22, 32, 46). Additional factors, including measurement techniques, device mechanics, body positioning, and transient physiological states, can further alter AOP values (11, 30–32, 41). While AOP captures some aspects of vascular variability, it does not fully reflect the metabolic, neuromuscular, or perceptual stress imposed during exercise, all of which are more directly linked to training adaptation (22, 32, 37). The effects of BFR training are influenced by multiple interacting factors, indicating that no single physiological measure can fully capture its complexity.

Personalization in BFR may therefore be more accurately conceptualized as an integrative process that accounts for multiple indicators of response rather than reliance on a single vascular metric. Defining personalization primarily through AOP may limit consideration of additional clinically relevant variables. It is well understood that pressure matters for both stimulus and safety, and that AOP represents a meaningful way to apply BFR with devices capable of full occlusion; both to help define an effective “floor” and a conservative “ceiling.” It is the conceptual positioning of AOP as the primary personalization parameter that merits continued examination. This perspective preserves the relevance of AOP while challenging its status as a comprehensive representation of individualized prescription.

In the absence of a singular physiological determinant of BFR dose, practical application may be guided by observable indicators of response. Perceptual measures such as rating of perceived exertion (RPE), limb discomfort, and tolerance remain among the most accessible and clinically relevant indicators. Performance-based markers, including fatigue progression and proximity to task failure, may further reflect the functional impact of restriction. Physiological responses such as heart rate may be monitored when clinically indicated, while measures such as muscle oxygen desaturation or blood lactate may provide additional insight, primarily within research or well-equipped environments. However, these measures are not routinely required for effective BFR application and have not been shown to consistently predict adaptive outcomes across individuals or conditions. Collectively, these considerations support a response-guided framework in which pressure is interpreted within the context of tolerance, performance, and progression, rather than reliance on any single physiological or numerical target.

## Translational validity

### The risk of methodological narrowing

Anchoring BFR training pressures to a measurable vascular threshold offers an appealing sense of objectivity, particularly within controlled research environments (10, 12). However, equating methodological precision with physiological necessity may overstate the role of AOP in determining effective BFR dose.

The growing emphasis on AOP has important implications for how BFR research is designed, interpreted, and translated. While AOP-based prescription offers clear advantages for internal validity, over-reliance on AOP-based models may inadvertently constrain experimental inquiry by privileging one form of personalization while discounting others that are clinically relevant and empirically supported. In real-world clinical, athletic, and fitness settings, BFR is frequently implemented without Doppler ultrasound or automated tourniquet systems, yet remains safe and effective when applied conservatively (3, 17, 25, 55, 56). Available evidence suggests that adaptive responses occur across a range of restrictive pressures rather than at a singular value, reflecting the internal physiological environment created by BFR rather than absolute pressure alone (1, 32, 37, 41).

Additionally, BFR training is fundamentally cumulative. Long-term outcomes depend on adherence, frequency, and tolerance across repeated sessions rather than optimization of any single bout (15, 24, 59). Methods that increase discomfort, complexity, or setup burden may inadvertently reduce BFR utilization, thereby offsetting any theoretical advantage offered by AOP.

From a research perspective, an exclusive focus on AOP risks narrowing the scope of inquiry by privileging specific devices and marginalizing alternative approaches that have demonstrated efficacy. Recent calls for improved reporting of cuff characteristics, exercise variables, and participant response, rather than fixation on pressure prescription alone, reflect growing recognition of this limitation (2, 14, 34).

It is equally important to acknowledge that non-AOP–based approaches are not without limitations. Fixed absolute pressures, limb circumference estimations, and elastic band applications may introduce variability in the degree of arterial inflow restriction achieved across individuals, as restriction magnitude is influenced by cuff width, limb size, systolic blood pressure, and device characteristics (10, 29, 30, 45). Without direct vascular assessment, the precise magnitude of arterial occlusion remains uncertain, and the potential for inadvertent supra-occlusive or sub-therapeutic pressures cannot be entirely excluded (10, 11, 33). Furthermore, variability in elastic material properties, band width, and application technique may influence pressure distribution and reproducibility across sessions and practitioners (35, 42, 53). Although practical and limb-based approaches have demonstrated efficacy across diverse populations, they similarly require careful application, symptom monitoring, and clinical reasoning to mitigate variability and optimize outcomes (2, 12, 17). Taken together, the variability observed across both AOP and non-AOP methodologies suggests that pressure prescription in BFR should be conceptualized as probabilistic and context-dependent rather than exact.

While individualized pressure selection is important, BFR safety reflects a broader system involving conservative programming, supervision, symptom monitoring, and population-specific screening. Importantly, many of these elements operate independently of AOP assessment. A comparative overview of the conceptual, methodological, and practical distinctions between AOP-based and non-AOP-based BFR pressure strategies are summarized in Table 1.

**Table 1:** Conceptual distinctions between AOP-based and Non-AOP-based BFR pressure strategies.

Domain	AOP-Based Strategy	Non–AOP-Based Strategy
Pressure Determination	Pressure prescribed as a percentage of individually measured arterial occlusion pressure (e.g., 40%–80% AOP)	Prescribed using conservative ranges, limb-size estimates, cuff scaling, or practitioner-guided adjustment without direct AOP measurement.
Physiological Rationale	Uses limb-specific AOP to standardize relative arterial restriction across individuals.	Assumes that partial venous restriction and metabolic stress can be achieved across a range of sub-occlusive pressures without requiring full arterial occlusion calibration
Adaptation Outcomes	Demonstrates improvements in strength, hypertrophy, and functional outcomes across clinical and athletic populations.	Demonstrates comparable improvements in strength, hypertrophy, and functional outcomes when training parameters are appropriate.
Precision & Variability	Greater theoretical precision; influenced by measurement conditions and device characteristics.	Less measurement-dependent; accommodates inherent physiological variability through monitoring and conservative dosing.
Safety Considerations	Low adverse event incidence when properly implemented; discomfort may increase with higher relative pressures.	Low adverse event incidence; risk primarily associated with excessive pressure or improper application.
Practical Implementation	Requires AOP-capable equipment and training.	Requires minimal equipment; adaptable to clinical, athletic, and remote settings.
Conceptual Trade-Off	Emphasizes pressure standardization.	Emphasizes accessibility and scalability.

Evidence suggests both AOP-based and non-AOP-based BFR pressure strategies demonstrate effective physiological and functional adaptations with low adverse event rates. Distinctions primarily relate to pressure determination methodology, theoretical precision, and practical implementation considerations.

## Discussion

### Toward an integrated framework for BFR pressure prescription

This narrative review examined the conceptual role of arterial occlusion pressure within blood flow restriction exercise, with particular attention to its necessity for safety, efficacy, and translational validity. Collectively, the available evidence supports AOP as a structured and defensible method for individualizing restrictive pressure, particularly within research environments where standardization enhances internal validity (10, 12, 18). However, current literature does not establish AOP as uniquely required for safe or effective implementation (16, 17, 26). Rather, adaptive responses to BFR appear to emerge from the interaction of mechanical, metabolic, perceptual, and programming variables that extend beyond resting occlusion thresholds alone (19, 28, 32, 37).

A central observation across outcome-based studies is that meaningful improvements in strength, hypertrophy, and functional performance occur across a wide range of sub-occlusive pressures and device types (12, 16, 18, 36, 47). This consistency suggests that while vascular calibration offers methodological clarity, adaptive mechanisms such as metabolite accumulation, intramuscular hypoxia, and fatigue-related motor unit recruitment are not strictly dependent on a singular relative AOP value (19, 22, 28, 45). Moreover, resting AOP is itself influenced by cuff width, limb position, tissue characteristics, and measurement technique (10, 11, 33), and may not fully reflect hemodynamic conditions present during dynamic exercise (32, 41).

Importantly, these observations do not invalidate AOP-based approaches. Rather, they clarify that AOP functions primarily as a reference point within a broader prescription framework. Its strengths lie in definable boundaries of restriction and methodological consistency, particularly when pneumatic systems capable of full occlusion are employed (10, 12). At the same time, alternative approaches (e.g., limb-based estimations and elastic systems) demonstrate efficacy across diverse settings, albeit with their own inherent variability (17, 42, 50). Both strategies therefore require clinical reasoning, symptom monitoring, and contextual adaptation (2, 12).

Framing pressure prescription as an exact determinant of stimulus may oversimplify the complexity of BFR physiology. Variability in cardiovascular responses, perceptual tolerance, cuff-device interactions, and exercise programming suggests that pressure selection operates within probabilistic boundaries rather than fixed thresholds (30, 35, 45). In this context, personalization may be more accurately conceptualized as an adaptive process incorporating vascular calibration, exercise intensity, patient tolerance, and longitudinal progression rather than reliance on a single metric (12, 35).

From a translational perspective, exclusive emphasis on AOP-based calibration may unintentionally narrow implementation pathways, particularly in settings where Doppler assessment or automated tourniquet systems are unavailable (3, 25). Conversely, dismissal of vascular measurement overlooks its value in research standardization and risk mitigation (10, 12). An integrated framework that recognizes the strengths and limitations of both methodologies may better reflect the multidimensional nature of BFR implementation.

Future investigations would benefit from direct comparative trials examining clinically meaningful outcomes across pressure-determination strategies, while systematically reporting cuff characteristics, pressure rationale, and participant tolerance (35, 14). Such work would clarify whether AOP provides distinct translational advantages or primarily serves as a procedural standardization tool. Without this context, comparisons between AOP and non-AOP based protocols may exaggerate differences attributable to methodological variation rather than true physiological effects.

**Figure 2:**
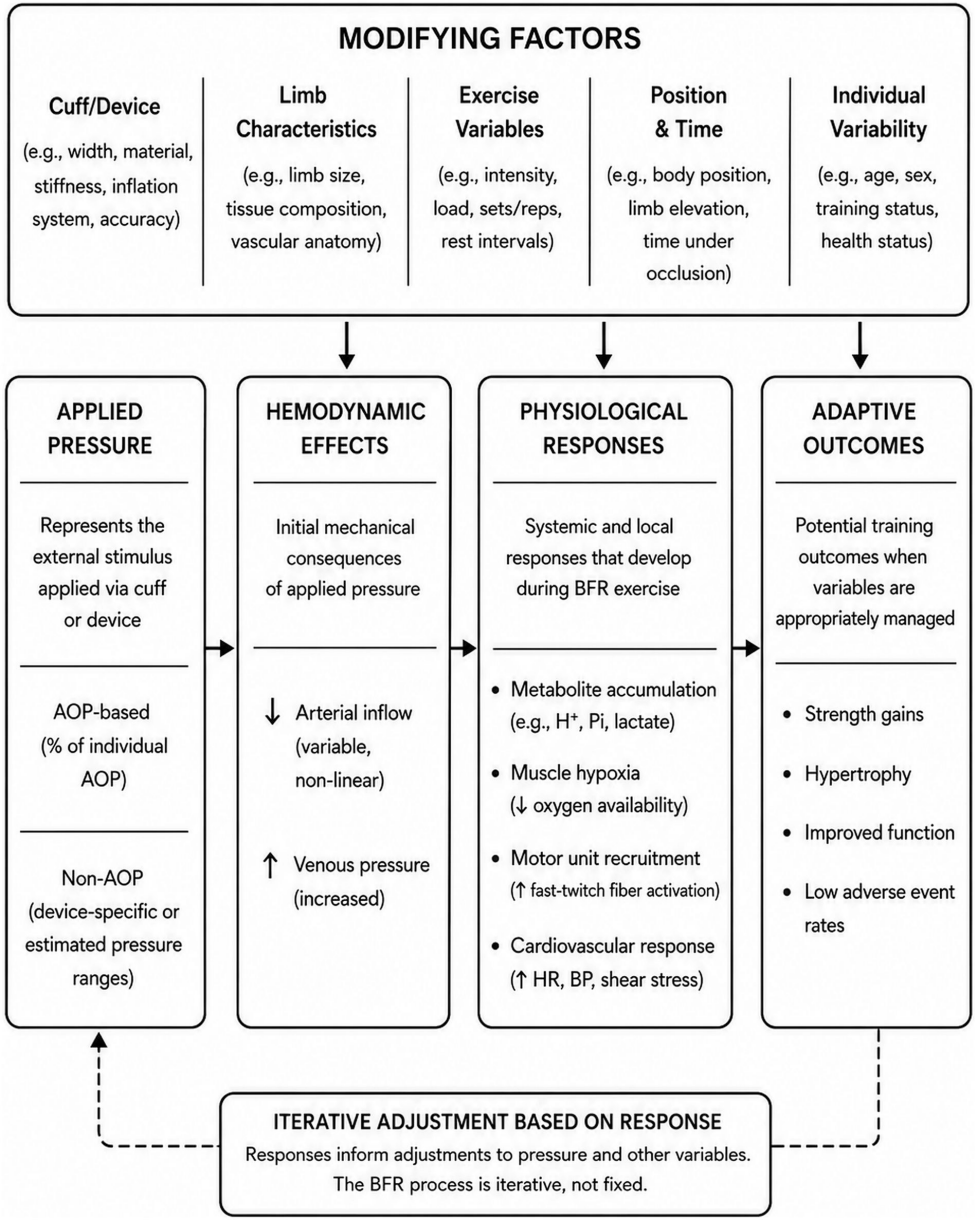
Conceptual framework illustrating how applied pressure interacts with modifying factors to influence physiological responses and adaptive outcomes during blood flow restriction (BFR) exercise. Applied pressure represents one input within a multifactorial system, with observed responses shaped by the interaction of individual, mechanical, and training-related variables.

In summary, current evidence suggests that AOP is valuable but not singularly determinative. Effective BFR implementation appears to arise from the coordinated interaction of applied pressure, device properties, exercise programming, and individual physiological response. Recognizing this interplay may support continued refinement of both research methodology and clinical practice without positioning any single approach as universally required. The multifactorial interaction between applied pressure, modifying variables, physiological responses, and adaptive outcomes illustrated in Figure 2 highlights the response-guided framework discussed throughout this manuscript.

## Conclusion

### Exact pressure precision may miss the point

Precise pressure calibration may represent one component of effective BFR implementation rather than its central determinant. Advocacy for AOP-based prescription reflects a thoughtful effort to enhance methodological rigor and safety in BFR research. Many high-quality trials rely on AOP-based prescription and have advanced the BFR literature. However, the available evidence does not support the notion that AOP is uniquely required to ensure safety, optimize adaptation, or individualize training responses. Meaningful physiological adaptations have been consistently demonstrated across a wide range of devices, pressures, and application strategies, many of which do not rely on individualized occlusion pressure measurements.

This observation does not diminish the value of AOP, nor does it suggest that pressure selection is inconsequential. Rather, it highlights the limitations of elevating any single metric as the defining determinant of BFR efficacy. Moreover, safety and adaptation appear to emerge from the interaction of applied pressure, exercise programming, device characteristics, and individual physiological and perceptual responses.

In this context, AOP should be viewed as a useful tool within a broader clinical and research toolkit; informative, but not indispensable. Safe and effective BFR implementation reflects the interaction between device characteristics, applied pressure, exercise programming, and individual response. As the field continues to evolve, progress may depend less on refining pressure precision and more on developing adaptive, multifactorial frameworks that account for the complexity of how physiological disruption is created, tolerated, and accumulated over time. This positioning allows AOP to retain methodological and clinical relevance without overstating its capacity to independently define physiological dose.

## References

[1] Freitas EDS Karabulut M Bemben MG. The evolution of blood flow restricted exercise. Front Physiol. (2021) 12:747759. 10.3389/fphys.2021.747759 34925056 PMC8674694

[2] Rolnick N Kimbrell K Cerqueira MS Weatherford B Brandner C. Perceived barriers to blood flow restriction training. Front Rehabil Sci. (2021) 2:697082. 10.3389/fresc.2021.697082 36188864 PMC9397924

[3] Scott BR Girard O Rolnick N McKee JR Goods PSR. An updated panorama of blood-flow-restriction methods. Int J Sports Physiol Perform. (2023) 18(12):1461–5. 10.1123/ijspp.2023-0135 37777193

[4] Jørgensen SL Kierkegaard-Brøchner S Bohn MB Aagaard P Mechlenburg I Schrøder HD. Effects of blood-flow restricted exercise versus conventional resistance training in musculoskeletal disorders—a systematic review and meta-analysis. BMC Sports Sci Med Rehabil. (2023) 15:141. 10.1186/s13102-023-00750-z 37880727 PMC10601135

[5] Wang X Chen Y Zhou L Hughes L Zhang Y Li Y. Effects of blood flow restriction training on bone metabolism: a systematic review and meta-analysis. Front Physiol. (2023) 14:1212927. 10.3389/fphys.2023.1212927 37621760 PMC10445948

[6] Yuan J Wu L Xue Z Xu G Wu Y. Application and progress of blood flow restriction training in improving muscle mass and strength in the elderly. Front Physiol. (2023) 14:1155314. 10.3389/fphys.2023.1155314 37035674 PMC10079911

[7] Alamri AA Aljuhani WS Alamri SA Aloraini AA Alamri SA. Blood flow restriction training in post-operative orthopedic rehabilitation: a systematic review and meta-analysis of randomized controlled trials. J. Musculoskelet. Surg. Res. (2025) 10(1):21–32. 10.25259/JMSR_365_2025

[8] Loenneke JP Thiebaud RS Abe T Bemben MG. Blood flow restriction pressure recommendations: the hormesis hypothesis. Med Hypotheses. (2014) 82(5):623–6. 10.1016/j.mehy.2014.02.023 24636784

[9] Lixandrão ME Ugrinowitsch C Laurentino G Libardi CA Aihara AY Cardoso FN. Effects of exercise intensity and occlusion pressure after 12 weeks of resistance training with blood-flow restriction. Eur J Appl Physiol. (2015) 115(12):2471–80. 10.1007/s00421-015-3253-2 26323350

[10] Jessee MB Buckner SL Dankel SJ Counts BR Abe T Loenneke JP. The influence of cuff width, sex, and race on arterial occlusion: implications for blood flow restriction research. Sports Med. (2016) 46(6):913–21. 10.1007/s40279-016-0473-5 26820301

[11] Hughes L Jeffries O Waldron M Rosenblatt B Gissane C Paton B. Influence and reliability of lower-limb arterial occlusion pressure at different body positions. PeerJ. (2018b) 6:e4697. 10.7717/peerj.4697 29736337 PMC5936068

[12] Patterson SD Hughes L Warmington S Burr J Scott BR Owens J. Blood flow restriction exercise: considerations of methodology, application, and safety. Front Physiol. (2019) 10:533. 10.3389/fphys.2019.00533 31156448 PMC6530612

[13] Das A Paton B. Is there a minimum effective dose for vascular occlusion during blood flow restriction training? Front Physiol. (2022) 13:838115. 10.3389/fphys.2022.838115 35464074 PMC9024204

[14] Hughes L Rolnick N Franz A Patterson SD Brandner CR Warmington SA. Blood flow restriction: methods and apparatus still matter. Br J Sports Med. (2025) 59(10):623–5. 10.1136/bjsports-2024-109365 39919806 PMC12171415

[15] Hughes DC Ellefsen S Baar K. Adaptations to endurance and strength training. Cold Spring Harbor Perspect Med. (2018) 8(6):a029769. 10.1101/cshperspect.a029769 PMC5983157 28490537

[16] Counts BR Dankel SJ Barnett BE Kim D Mouser JG Mattocks KT. Influence of relative blood flow restriction pressure on muscle adaptations. Med Sci Sports Exerc. (2016) 48(5):911–5. 10.1002/mus.24756

[17] Scott BR Loenneke JP Slattery KM Dascombe BJ. Exercise with blood flow restriction: an updated evidence-based approach for enhanced muscular development. Sports Med. (2015) 45(3):313–25. 10.1007/s40279-014-0288-1 25430600

[18] Clarkson MJ May AK Warmington SA. Is there rationale for the cuff pressures prescribed for blood flow restriction exercise? A systematic review. Scand J Med Sci Sports. (2020) 30:1318–36. 10.1111/sms.13676 32279391

[19] Loenneke JP Abe T Wilson JM Thiebaud RS Fahs CA Rossow LM. Blood flow restriction: how does it work? Front Physiol. (2012) 3:392. 10.3389/fphys.2012.00392 23060816 PMC3463864

[20] Loenneke JP Fahs CA Rossow LM Thiebaud RS Mattocks KT Abe T. Blood flow restriction pressure recommendations: a tale of two cuffs. Front Physiol. (2013) 4:249. 10.3389/fphys.2013.00249 24058346 PMC3767914

[21] Loenneke JP Wilson JM Marín PJ Zourdos MC Bemben MG. Rehabilitation of an osteochondral fracture using blood flow restricted exercise: a case review. J Bodyw Mov Ther. (2013) 17(1):42–5. 10.1016/j.jbmt.2012.04.006 23294682

[22] Crossley KW Porter DA Ellsworth J Caldwell T Feland JB Mitchell U. Effect of cuff pressure on blood flow during blood flow-restricted rest and exercise. Med Sci Sports Exerc. (2020) 52(3):746–53. 10.1249/MSS.0000000000002156 31568024

[23] de Queiros VS Rolnick N Neto GR de França IM de Almeida MB da Silva-Grigoletto ME. Body position and cuff size influence lower limb arterial occlusion pressure and its predictors: implications for standardizing the pressure applied in training with blood flow restriction. Front Physiol. (2024) 15:1446963. 10.3389/fphys.2024.1446963 39189031 PMC11345145

[24] Hughes L Paton B Rosenblatt B Gissane C Patterson SD. Blood flow restriction training in clinical musculoskeletal rehabilitation: a systematic review and meta-analysis. Br J Sports Med. (2017) 51(13):1003–11. 10.1136/bjsports-2016-097071 28259850

[25] Lorenz DS Bailey L Wilk KE Mangine RE Head P Grindstaff TE. Blood flow restriction training. J Athl Train. (2021) 56(9):937–44. 10.4085/418-20 34530434 PMC8448465

[26] Dancy ME Alexander AS Abbas MJ Rolnick N Alder KD Lu Y. No differences in exercise performance, perceptual response, or safety were observed among 3 blood flow restriction devices. Arthrosc Sports Med Rehabil. (2023) 5(6):100822. 10.1016/j.asmr.2023.100822 38058769 PMC10696247

[27] Carter DM Chatlaong MA Miller WM Benton JB Jessee MB. Comparing the acute responses between a manual and automated blood flow restriction system. Front Physiol. (2024) 15:1409702. 10.3389/fphys.2024.1409702 38948082 PMC11211589

[28] Loenneke JP Fahs CA Rossow LM Sherk VD Thiebaud RS Abe T. Effects of cuff width on arterial occlusion: implications for blood flow restricted exercise. Eur J Appl Physiol. (2012) 112:2903–12. 10.1007/s00421-011-2266-8 22143843 PMC4133131

[29] Loenneke JP Allen KM Mouser JG Thiebaud RS Kim D Abe T. Blood flow restriction in the upper and lower limbs is predicted by limb circumference and systolic blood pressure. Eur J Appl Physiol. (2015) 115(2):397–405. 10.1007/s00421-014-3030-7 25338316

[30] Hunt JEA Stodart C Ferguson RA. The influence of participant characteristics on the relationship between cuff pressure and level of blood flow restriction. Eur J Appl Physiol. (2016) 116(8):1421–32. 10.1007/s00421-016-3399-6 27235157 PMC4911379

[31] Ingram JW Dankel SJ Buckner SL Counts BR Jessee MB Mattocks KT. The influence of time on determining blood flow restriction pressure. Journal of Science and Medicine in Sport. (2017) 20(8):777–80. 10.1016/j.jsams.2016.11.013 28131507

[32] Mouser JG Ade CJ Black CD Bemben DA Bemben MG. Brachial blood flow under relative levels of blood flow restriction is decreased in a nonlinear fashion. Clin Physiol Funct Imaging. (2018) 38(3):425–30. 10.1111/cpf.12432 28402045

[33] Sieljacks P Knudsen L Wernbom M Vissing K. Body position influences arterial occlusion pressure: implications for the standardization of pressure during blood flow restricted exercise. Eur J Appl Physiol. (2018) 118(2):303–12. 10.1007/s00421-017-3770-2 29196847

[34] Stray-Gundersen S Wooten S Tanaka H. Walking with leg blood flow restriction: wide-rigid cuffs vs. Narrow-elastic bands. Front Physiol. (2020) 11:568. 10.3389/fphys.2020.00568 32547424 PMC7273976

[35] Rolnick N Kimbrell K de Queiros V. Beneath the cuff: often overlooked and under-reported blood flow restriction device features and their potential impact on practice—a review of the current state of the research. Front Physiol. (2023) 14:1089065. 10.3389/fphys.2023.1089065 37064884 PMC10099250

[36] Minniti MC Statkevich AP Kelly RL Rigsby VP Exline MM Rhon DI. The safety of blood flow restriction training as a therapeutic intervention for patients with musculoskeletal disorders: a systematic review. Am J Sports Med. (2020) 48(7):1773–85. 10.1177/0363546519882652 31710505

[37] Hornikel B Saffold KS Mota JA Esco MR Fedewa MV Wind SA. Acute blood flow responses to varying blood flow restriction pressures in the lower limb. Int J Exerc Sci. (2023) 16(2):118–28. 10.70252/IRPL4226 37114195 PMC10124717

[38] Citherlet T Willis SJ Chaperon A Millet GP. Differences in limb blood flow between two types of blood flow restriction cuffs: a pilot study. Front Physiol. (2022) 13:931270. 10.3389/fphys.2022.931270 35957986 PMC9360536

[39] Ilett MJ Rantalainen T Keske MA May AK Warmington SA. The effects of restriction pressures on the acute responses to blood flow restriction exercise. Front Physiol. (2019) 10:1018. 10.3389/fphys.2019.01018 31456694 PMC6700307

[40] Cerqueira MS Lira M Mendonça Barboza JA Burr JF Wanderley e Lima TB Maciel DG. Repetition failure occurs earlier during low-load resistance exercise with high but not low blood flow restriction pressures: a systematic review and meta-analysis. J Strength Cond Res. (2021). (Advance online publication). 10.1519/JSC.0000000000004093 34319945

[41] Mladen SPS Forbes SPA Zedic AK England VS Drouin PJ Tschakovsky ME. Leg blood flow during exercise with blood flow restriction: evidence for and implications of compensatory cardiovascular mechanisms. J Appl Physiol. (2025) 138(2):492–507. 10.1152/japplphysiol.00772.2024 39818968

[42] Aniceto RR da Silva Leandro L. Practical blood flow restriction training: new methodological directions for practice and research. Sports Med Open. (2022) 8(1):87. 10.1186/s40798-022-00475-2 35763185 PMC9240154

[43] Fleming AR MacDonald HV Buckner SL Winchester LJ. Lower limb blood flow occlusion increases systemic pressor response without increasing brachial arterial blood flow redistribution in women. Clin Physiol Funct Imaging. (2024) 44(4):285–96. 10.1111/cpf.12873 38402408

[44] Yamada Y Hammert WB Kataoka R Song JS Kang A Kassiano W. The role of the muscle metaboreflex on cardiovascular responses to submaximal resistance exercise with different pressures and modes of blood flow restriction. Appl Physiol Nutr Metab. (2025) 50:1–9. 10.1139/apnm-2024-0383 39899811

[45] Mouser JG Dankel SJ Jessee MB Mattocks KT Buckner SL Counts BR. A tale of three cuffs: the hemodynamics of blood flow restriction. Eur J Appl Physiol. (2017) 117(7):1493–9. 10.1007/s00421-017-3644-7 28501908

[46] Jessee MB Mattocks KT Buckner SL Dankel SJ Mouser JG Abe T. Mechanisms of blood flow restriction: the New Testament. Tech Orthop. (2018) 33(2):72–9. 10.1097/BTO.0000000000000252

[47] Lixandrão ME Ugrinowitsch C Berton R Vechin FC Conceição MS Damas F. Magnitude of muscle strength and mass adaptations between high-load resistance training versus low-load resistance training associated with blood-flow restriction: a systematic review and meta-analysis. Sports Med. (2018) 48(2):361–78. 10.1007/s40279-017-0795-y 29043659

[48] Moghaddam M Rabel MC Werner T Rolnick N. Effects of autoregulated and non-autoregulated blood flow restriction on vastus medialis oblique responses during low-load resistance exercise. Front Sports Act Living. (2025) 7:1712606. 10.3389/fspor.2025.1712606 41458102 PMC12740858

[49] Akcay N Rolnick N Keskin K Kamis O Koremezli Keskin N Basdemirci O. Autoregulation during blood flow restricted exercise offers no additional benefit on thigh muscle hypertrophy and strength adaptations in trained participants: a randomized within-subject 8-week trial. Front Physiol. (2026). (Advance online publication). 10.3389/fphys.2026.1772708 41810114 PMC12967994

[50] Luebbers PE Witte EV Oshel JQ Butler MS. Effects of practical blood flow restriction training on adolescent lower-body strength. Journal of Strength and Conditioning Research. (2019) 33(10):2674–83. 10.1519/JSC.0000000000002302 29084094

[51] Behringer M Behlau D Montag JCK McCourt ML Mester J. Low-intensity sprint training with blood flow restriction improves 100-m dash. Journal of Strength and Conditioning Research. (2017) 31(9):2462–72. 10.1519/JSC.0000000000001746 27941491

[52] Held S Behringer M Donath L. Low intensity rowing with blood flow restriction over 5 weeks increases v˙O2max in elite rowers: a randomized controlled trial. Journal of Science and Medicine in Sport. (2020) 23(3):304–8. 10.1016/j.jsams.2019.10.002 31672481

[53] Abe T Mouser JG Dankel SJ Bell ZW Buckner SL Mattocks KT. A method to standardize the blood flow restriction pressure by an elastic cuff. Scand J Med Sci Sports. (2019) 29(3):329–35. 10.1111/sms.13340 30468528

[54] McEwen JA Owens JG Jeyasurya J. Why is it crucial to use personalized occlusion pressures in blood flow restriction rehabilitation? J Med Biol Eng. (2018) 39:173–7. 10.1007/s40846-018-0397-7

[55] Takarada Y Takazawa H Sato Y Takebayashi S Tanaka Y Ishii N. Effects of resistance exercise combined with moderate vascular occlusion on muscular function in humans. J Appl Physiol. (2000) 88(6):2097–106. 10.1152/jappl.2000.88.6.2097 10846023

[56] Nakajima T Kurano M Iida H Takano H Oonuma H Morita T. Use and safety of KAATSU training: results of a national survey. Int J KAATSU Train Res. (2006) 2(1):5–13. 10.3806/ijktr.2.5

[57] Yasuda T Meguro M Sato Y Nakajima T. Use and safety of KAATSU training: Results of a national survey in 2016. Int J KAATSU Train Res. (2017) 13(1):1–9. 10.3806/ijktr.13.1

[58] Swain PSP McEwen JM Lai TL Hughes LH. Tourniquet cuff pressure during blood flow restriction exercise. Front Sports Act Living. (2025) 7:1582387. 10.3389/fspor.2025.1582387 40969978 PMC12441828

[59] Slysz J Stultz J Burr JF. The efficacy of blood flow restricted exercise: a systematic review and meta-analysis. J Sci Med Sport. (2016) 19(8):669–75. 10.1016/j.jsams.2015.09.005 26463594

